# Sol-Gel Immobilisation of Lipases: Towards Active and Stable Biocatalysts for the Esterification of Valeric Acid

**DOI:** 10.3390/molecules23092283

**Published:** 2018-09-06

**Authors:** Soledad Cebrián-García, Alina M. Balu, Araceli García, Rafael Luque

**Affiliations:** 1 Organic Chemistry Department, University of Cordoba, Campus de Rabanales, Edificio Marie Curie (C-3), Ctra. Nacional IV-A, Km 396, E14014 Cordoba, Spain; b02cegas@uco.es (S.C.-G.); qo2balua@uco.es (A.M.B.); qo2ganua@uco.es (A.G.); 2 Scientific Centre for Molecular Design and Synthesis of Innovative Compounds for Medicine, Peoples Friendship University of Russia (RUDN University), 6 Miklukho-Maklaya str., 117198 Moscow, Russia

**Keywords:** sol-gel biosilicification, esterification, conventional heating, microwaves

## Abstract

Alkyl esters are high added value products useful in a wide range of industrial sectors. A methodology based on a simple sol-gel approach (biosilicification) is herein proposed to encapsulate enzymes in order to design highly active and stable biocatalysts. Their performance was assessed through the optimization of valeric acid esterification evaluating the effect of different parameters (biocatalyst load, presence of water, reaction temperature and stirring rate) in different alcoholic media, and comparing two different methodologies: conventional heating and microwave irradiation. Ethyl valerate yields were in the 80–85% range under optimum conditions (15 min, 12% *m*/*v* biocatalyst, molar ratio 1:2 of valeric acid to alcohol). Comparatively, the biocatalysts were slightly deactivated under microwave irradiation due to enzyme denaturalisation. Biocatalyst reuse was attempted to prove that good reusability of these sol-gel immobilised enzymes could be achieved under conventional heating.

## 1. Introduction

In recent years, the production of high added value products from biomass has become an important subject of study in many scientific fields, as biomass encompasses a wide range of organic materials characterized by its heterogeneous origin and composition. For instance, sewage sludge, urban solid organic wastes and other residues generated from industrial processes [1] and other human activities can be also considered as biomass sources. Moreover, biomass is considered the largest worldwide renewable source of carbon, as it can be originated through biological pathways. Currently, biomass is the fourth largest worldwide energy resource, becoming potentially a sustainable source of valuable chemicals through various transformation pathways, among which enzymatic conversion should be highlighted [2].

Carboxylic acids are common components of effluents and sub-streams from several industrial procedures. While these compounds do not present interesting applications, their esters have numerous uses in the food, cosmetic and pharmaceutical industries, representing a huge demand estimated to be worth $23.5 billion in 2014 [3,4]. The main issues of alkyl esters are their very limited natural supply, and their chemical synthesis via catalytic procedures requires severe reaction conditions with a quite low selectivity of inorganic catalysts [5,6]. In this sense, biological catalysts are a promising alternative in the production of alkyl esters under moderate and mild conditions [3,6,7]. They show a more efficient and ecological alternative to the use of traditional inorganic catalysts for biomass valorisation because of their good performance at environmentally friendly conditions, lower temperature and pH values, and fewer by-products because of their regioselectivity and stereoselectivity [8]. Therefore, it is not surprising that biocatalysed processes have been steadily growing over recent years, with clear outlooks indicating an increasing trend for enzyme commercialisation in the global market.

The use of these biocatalysts is not well established at industrial scale due to two main drawbacks: their limited stability, being not compatible with continuous production processes [9], and the complication of the substrates/products separation from the reaction medium, avoiding their reuse [10]. In order to overcome these important issues, research on enzyme immobilization is increasingly relevant, allowing the development of several biocatalytic industrial processes towards economically profitable applications. A simple immobilization protocol implemented in our group, denoted as biosilicification, has been proved to improve enzyme stability in organic media, enhancing catalytic efficiency at the same time [9]. The enzyme biosilicification process is based on a simple sol-gel synthetic procedure in which a silica precursor (e.g., tetraethylorthosilicate) is dropwise added to the enzyme and hydrolysed, in order to partially encapsulate the enzyme by a silica layer [9]. Although, most of the research works have focused on the immobilization of hydrolytic enzymes, such as lipases, more recent studies have employed the same procedure in laccases with similar excellent results [9,11]. Furthermore, due to immobilization, the recovery and subsequent reuse of these biocatalysts is a favoured characteristic, reducing the final production costs when compared with the use of free enzymes [11].

**Figure 1:**
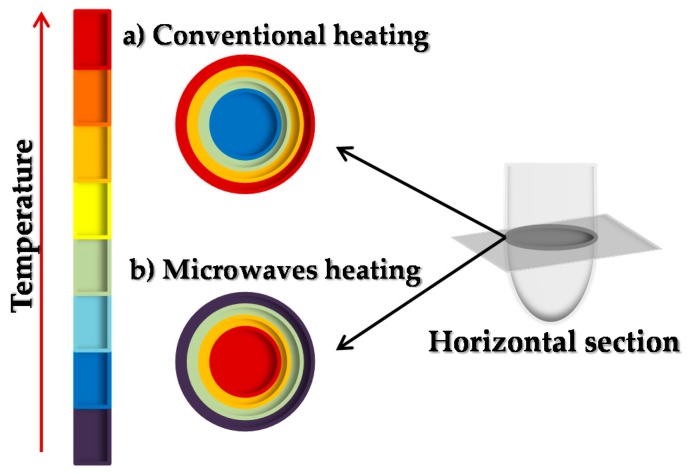
Temperature profiles provided by (**a**) conventional heating or (**b**) microwave-assisted processes.

Conventional heating (CH) methods have been reported as slow and inefficient energy transfer production pathways due to the disadvantage that they present on the adequate convection currents transference and the low thermal conductivity of the materials that they need to penetrate [12,13]. These issues result in a higher temperature in the reaction tube than in the reaction medium, promoting a reverse temperature gradient. This problem can be solved by using microwave (MW)-assisted heating procedures (Figure 1), as the energy is directly transferred to reactive species by so-called “molecular heating” processes, promoting more selective transformations that are not possible to achieve by conventional heating [13,14,15]. MW radiation, as any electromagnetic radiation, is constituted by an electric field and a magnetic field that propagates perpendicularly, although only the electric field transfers the energy, through interaction of dipoles and ionic conduction, which leads to the heating of the substances [13,16]. It is completely different from what happens in a conventional heating reactor, where the heating takes place by conduction, irradiation and convection [13]. Conventional heating also entails longer reaction times [17] in comparison with MW reactors but it leads to cost and energy savings because of an easy and fast capacity to scale up in different industries.

Esterification reactions can be defined as the transformation of carboxylic acids or their derivatives into esters. The kinetic and process behaviour of this important type of reactions have been thoroughly investigated since Berthelot and Gilles [18] began the first studies in 1862. The most widely employed method comprises the direct esterification of carboxylic acid with alcohols in the presence of acid catalysts. However, conventional acid catalysts during esterification reactions exhibit problems associated with the generation of secondary reactions, corrosion of equipment, costly purification procedures, and long reaction times [19]. Therefore, the need for an esterification reaction system providing cleaner and shorter routes to synthesize a wide variety of industrial products, such as valeric acid esters, results in being essential. Considering this, the aim of the present work was to optimize the catalytic esterification of valeric acid to ethyl valerate (marketed as green apple flavour) by using sol-gel biosilicified enzymes and comparing the performance of two different methodologies: conventional heating and MW-assisted processes. Moreover, different experiments with metal containing biocatalysts were carried out in order to improve their recovery and subsequent reuse [20].

## 2. Results and Discussion

The esterification reaction was carried out using valeric acid as substrate, as carboxylic acid, and different short chain alcohols such as methanol (MeOH), ethanol, isopropanol (iPrOH) and 1-butanol (BuOH) as reactive media. The hydrolytic free and immobilized enzyme, *Candida antarctica* lipase B (CalB), was employed in order to study the efficiency of the encapsulation or biosilicification procedure, conducted by CH and MW methodologies. The immobilized lipase was previously characterized by our group using different techniques such as Brunauer-Emmett-Teller surface area analysis (BET), thermogravimetric analysis (TGA) and X-Ray Photoelectron Spectroscopy (XPS) [20]. The reported results indicated that the biocatalyst started being a non-porous material, becoming highly porous after successive reuses due to the progressive loss of organic compounds. Despite these protein losses, the biocatalyst still retained significant amounts of lipase, remaining highly active during the investigated esterification reactions. These relevant results further supported the high stability of the immobilised enzymes compared with the free enzyme, particularly under the investigated conditions [20].

### 2.1. Protein Concentration and Enzymatic Activity

For each experiment 12% *m*/*v* of biocatalyst was introduced in the mixture of reaction, immobilized or free form, presenting an enzymatic activity of 592 U/g_enzyme_ or 656 U/g_enzyme_, respectively (Table 1). As expected, the fresh free enzyme showed a higher value than synthetized biosilicified lipase. This was due to the enzyme immobilization process that hid many active centres inside the silica crust. Leaching was expected to take place after reuse, considering the methodology and reaction conditions at which the immobilized enzyme was be exposed. On the other hand, the support does not show any enzymatic activity which identifies the lipase as responsible for the esterification reaction, in this procedure.

### 2.2. Determination of Optimal Conditions

**Figure 2:**
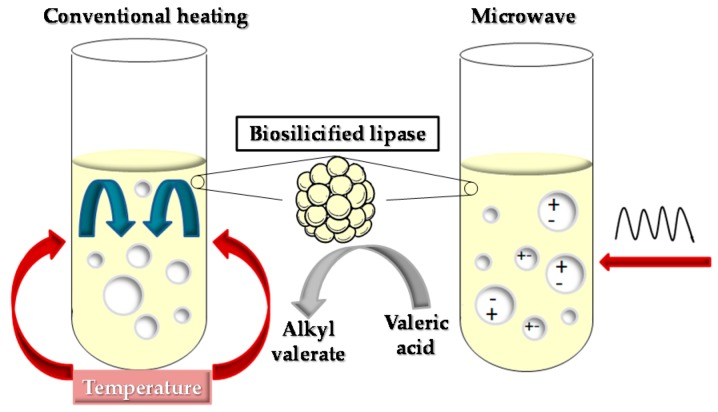
Conventional heating (CH) and microwave (MW)-assisted heating methodologies for the esterification of valeric acid catalysed by biosilicified enzymes.

The evaluation of the effect of different parameters (presence of water, biocatalyst load, stirring rate and reaction temperature) on the valeric acid esterification behaviour was carried out by means a set of experiments conducted using CH procedures for the subsequent evaluation of the conversion to ethyl acetate by using both CH and MW methodologies (Figure 2).

#### 2.2.1. Presence of Water during Esterification Reaction

The presence of water (distilled water (DW)% *m*/*v*) in the reaction has been reported to favour enzyme-substrate interaction in biocatalysed reactions. In the present study, this parameter did not significantly affect the conversion of valeric acid (Table 2).

None of the conversions achieved by the prepared biocatalyst or the specific activity demonstrated presented remarkable differences between different percentages of DW studied. However, further investigations are needed [15,21] for systems catalysed by sol-gel biosilicified enzymes.

#### 2.2.2. Influence of Biocatalyst Load

**Figure 3:**
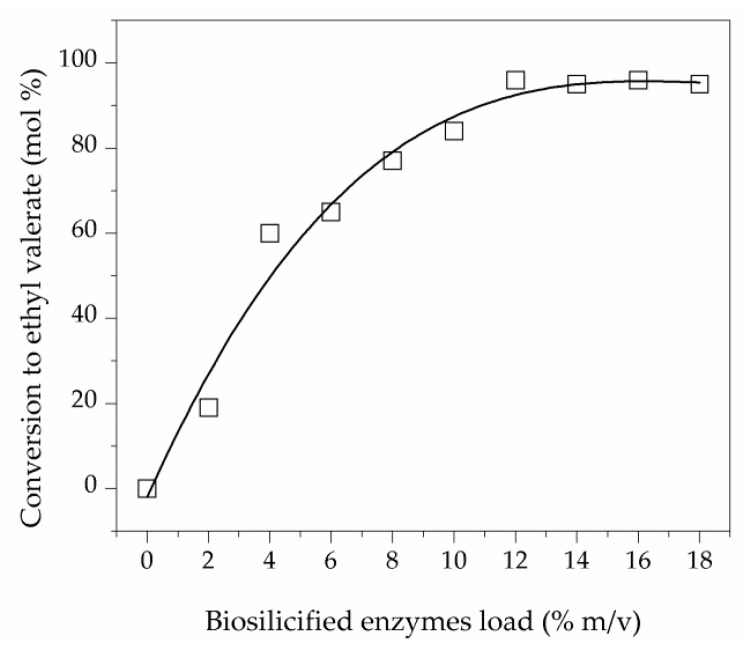
Effect of amount of biosilicified enzyme on the conversion of valeric acid to ethyl valerate (study conditions: molar ratio 1:2 of valeric acid to EtOH, 1000 rpm, 40 °C, 2 h in CH batch reactor).

The optimal concentration of sol-gel biosilicified enzyme in the reaction was determined by a set of experiments using the CH procedure (Figure 3). 

A 12% *m*/*v* of biocatalyst load provided a maximum conversion of 96% with 100% of reaction selectivity, and no difference could be observed when higher amount of catalyst was added. Such an enzyme concentration was consequently considered for subsequent experiments.

#### 2.2.3. Influence of Stirring Rate

**Figure 4:**
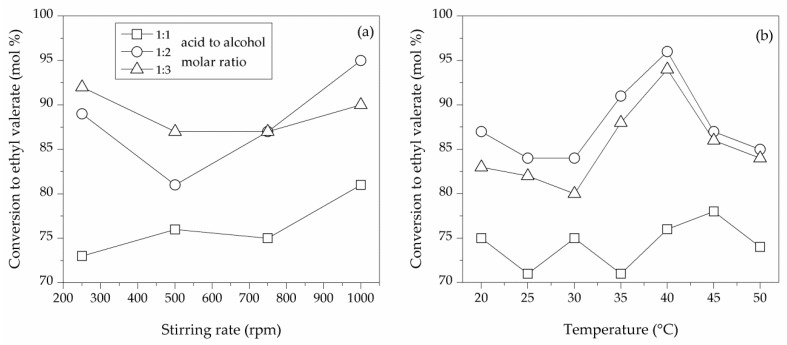
Effect of (**a**) stirring rate and (**b**) reaction temperature on the conversion of valeric acid to ethyl valerate by CH procedures.

Mass transfer limitations can affect the conversion efficiency of reactions, especially related to heterogeneous catalysis [22]. Consequently, appropriate and efficient stirring is needed for uniform mixing and contact between reactants in the reaction medium. In the present study, valeric acid conversions between 73% and 95% were obtained for different acid-to-alcohol molar ratios, not being significantly influenced by the stirring rate (Table 3). However, the higher conversion was achieved at high stirring rates for medium and high acid concentrations (Figure 4a). Regarding enzymatic activities, better values were obtained at higher stirring rate (501 ± 13 U/g_biocatalyst_ at 100 rpm).

#### 2.2.4. Influence of Reaction Temperature

As reported in previous articles, temperature is key factor in the proper operation of biological molecules such as enzymes [23]. Previous studies [24] reported 70 °C as a critical temperature for the stability of enzymes. Furthermore, temperatures higher than 50 °C have been reported to promote denaturation in the native state of proteins, with a complete loss of enzymatic activity. Comparatively, lower temperatures may lead to aggregation in the reaction medium and a decrease in catalytic efficiency [23]. Therefore, the study of the temperature in the optimization of CH esterification of valeric acid by lipases is decisive. In the present work, immobilized and free enzymes were tested up to 50 °C. Interestingly, the highest conversion rate (96%, 100% selectivity to ethyl valerate) was obtained at 40 °C and with a 1:2 acid-to-alcohol molar ratio after 2 h (Table 4). Experiments conducted at different acid-to-alcohol molar ratios and temperatures indicated that moderate to very good ethyl valerate yields could be obtained (71–96% conversion at complete ethyl valerate selectivity) even at high valeric acid concentration (Figure 4b).

On the other hand, the prepared biocatalysts present the optimum activity values temperature in a range between 35–45 °C and at 1:2 acid-to-alcohol molar ratio. Immobilized catalysts were stirred in aqueous solution for 2 h in a range of different temperatures. Table 4 shows a substantial decrease in enzymatic activity with temperature under innocuous conditions, which results a determining factor in case of biocatalytic processes.

### 2.3. Effect of Alcohol Type on Lipase Catalysed Esterification of Valeric Acid

#### 2.3.1. Production of Ethyl Valerate by Conventional Heating (CH) Procedures

Table 5 summarises the main results from a set of experiments conducted under optimised reaction conditions with conventional heating. Similarly, experiments performed under CH in the absence of biocatalyst (blank) or using SiO_2_ support (SiO_2_ crust without enzyme) did not lead to valeric acid conversion for all tested alcohols. Comparably, the highest conversion rate (95%, 100% selectivity to ethyl valerate) was obtained using a 12% *m*/*v* of biosilicified enzyme and a 1:2 acid-to-alcohol molar ratio after 2 h (Table 5, entry 14). Product yields obtained using a 12% *m*/*v* free lipase and different acid-to-alcohol ratios were about 80% (Table 5, entries 9–11). Moreover, reactions conducted with MeOH as the alcohol provided negligible activities in the systems (Table 5, entry 12), in good agreement with previous results from the group [9,11,25]. MeOH seems to be toxic for some enzymes, especially CalB, and therefore they do not reach significant conversion in these alcoholic systems. Previous studies reported that CalB, even if very stable in a wide range of organic solvents, is rapidly deactivated in the presence of MeOH at molar ratios much lower than the theoretical optimum for the conversion reaction [26]. However, the existence of specific activity after enzyme incubation in MeOH medium indicated that the lack of valeric acid conversion was not only due to denaturation mechanisms, but this alcohol specifically decreases the hydrolytic activity of CalB (Table 5, entry 12), as reported in previous works [27]. The specific activity of the free enzyme after the esterification reaction (600 ± 12 U/g_biocatalyst_) resulted comparably lower to that found for the fresh free enzyme (656 ± 14 U/g_biocatalyst_ in Table 1) after enzyme incubation in the presence of EtOH. This indicated certain weathering of the biocatalyst, as several active centres could be deactivated by denaturing processes. However, for the immobilized enzyme the specific activity increased from 592 ± 13 (see Table 1) to 640 ± 17 U/g_biocatalyst_ after its use. During the immobilization process, many active centres could be hidden inside the silica layer, being partially washed and released after the first use of the biocatalyst, with the subsequent increase in specific activity.

Finally, the use of other alcohols (such as iPrOH and BuOH) mediated conversions to alkyl valerate of about 90% (Table 5, entries 17, 20) in a 1:2 acid:alcohol molar ratio, but in general with significantly lower yields than when EtOH was used. Moreover, considering specific activity values, CalB appeared to be more stable in ethanol than in iPrOH and BuOH because after the biocatalyst incubation in the presence of iPrOH or BuOH, the remaining specific activity of the immobilized enzyme was much lower compared to the catalyst incubated in the presence of EtOH. During esterification experiments using these two alcohols, the biocatalyst behaviour was different, and certain deposition/precipitation of particles was observed. Thus, partial steric impedance could occur due lower solubility and explain the decrease of the catalyst’s specific activities in these alcoholic media. On the other hand, the support does not show any enzymatic activity which identifies the lipase as responsible for the esterification reaction, in this procedure. The effect of the alcohol used on the catalyst is studied in Table 5. As it could be expected, the lipase incubated with ethanol maintains the highest enzymatic activity 640 U/g_enzyme_.

The stability and reusability of the enzyme under the investigated CH conditions was subsequently tested (Table 6). Results pointed out that the biosilicified lipase keeps its conversion capacity in the ethanol stable. This is not so for iPrOH and BuOH due to their toxicity [28]. Selectivity to alkyl valerate was fully preserved after reuses in each case. Comparably, the free enzyme provided 82% conversion at complete selectivity in the first run, followed by a significant reduction of conversion of the enzyme (<50% after 1 reuses, R1), most probably due to denaturalisation under temperature.

#### 2.3.2. Production of Ethyl Valerate Using Microwave (MW) Irradiation

Compared to the CH procedure, blank runs (in the absence of biocatalyst) including those with the silica support (SiO_2_ crust without enzyme) did not provide any valeric acid conversion (Table 7).

The highest conversion rate (82%, with 100% selectivity to ethyl valerate) was obtained using a 12% *m*/*v* of biosilicified lipase and a 1:2 acid:alcohol molar ratio after 1 h (Table 7, entry 14). Product yields obtained using a 12% *m*/*v* of free lipase and different acid-to-alcohol ratios were found to be higher than 70% (Table 7, entries 9–11). Moreover, reactions conducted with MeOH as alcohol provided negligible activities in the systems (Table 7, entry 12). Finally, the use of other alcohols (such as iPrOH and BuOH) mediated conversions to alkyl valerate of 40% in iPrOH and 80% in BuOH (Table 7, entries 17, 20) in a 1:2 acid-to-alcohol molar ratio. This technique aimed to simplify the esterification procedure as the reaction energy is transferred directly to reactive species [13,16]. In the case of enzymatic activity, it could be said that CH is an efficient procedure where biocatalyst remains stable without loss of their catalytic efficiency. It was also found that CH methodology increased the catalytic efficiency under the pressure- and temperature-mild conditions required for lipases, whereas a reaction conducted using MW as the heating source resulted quite focused on the reactive species, interfering with the thermal stability of the proteins.

In Table 8, the results of the reaction investigated under optimised conditions throughout the present work are shown. As observed, after 15 min of reaction the conversion to ethyl valerate was higher than 80% with a 100% of selectivity in both cases. These values kept almost equal for longer MW-assisted reactions, whereas higher yields were obtained for CH conducted procedures (90% at 30 and 60 min). On the other hand, a clear increase of the enzymatic activity with the reaction time was observed for both CH and MW methodologies.

Although these two methodologies offered good results for the valeric acid esterification reaction, and even if MW could result in a more efficient heating procedure, CH still provides a more environmentally sustainable, economically profitable and easily scaled-up procedure, due to the lower costs related with the energy requirements that it provides. Conditions reached during MW-assisted reaction were too severe for lipases, despite their high stability reached under sol-gel biosilicification, and significantly lower specific activities were observed in MW reactions in comparison to those observed for CH.

The stability and reusability of the enzymes used under the above investigated MW conditions were subsequently tested (Table 9). Results pointed out that the biosilicified lipases gradually lost their conversion capacity when reuses were performed in EtOH medium (from 82 to 35% after 3 uses), but importantly the enzymes were not fully denaturalised after several cycles under the investigated conditions. Lipases require quite moderate reaction conditions that cannot be established in microwaves due to the rapid heating to which the molecules in reaction are subjected [16].

## 3. Materials and Methods

### 3.1. Materials

The reagents Tetraethoxysilane 98% (TEOS), n-dodecylamine 98%, buffer solution of enzyme *Candida antarctica* lipase B 5000 LU/g (CalB), titanium dioxide 99.5%, p-nitrophenyl butirate 98% (pNPB), valeric acid 99% and Bradford reagent were purchased from Sigma Aldrich. Ethanol 98% (EtOH), methanol 99.5% (MeOH), isopropanol 99.5% (iPrOH), 1-butanol 99.5% (BuOH), acetonitrile 99.9% and Cobalt (II) chloride hexahydrates 98% were purchased from Panreac and used without further purification.

### 3.2. Enzyme Biosilicification

According to the protocol previously described [29], TEOS (20.80 g, 0.10 mol) was added to a continuously stirred solution containing n-dodecylamine (5.10 g, 0.03 mol), 50 g of acetonitrile and 50 g CalB buffer solution at room temperature. For the synthesis of immobilized enzymes with two different metal precursors, after addition of the acetonitrile and n-dodecylamine, titanium dioxide and cobalt (II) chloride hexahydrates (1% wt.) were added under continuous stirring, before the CalB buffer solution and TEOS addition. The resulting solutions presented a visible solid precipitate after a few minutes, so they were further stirred for another 3 h. Finally, the synthetized biocatalysts were filtered, twice washed with ethanol, and dried at room temperature for 24 h. For the preparation of the SiO_2_ reference support, the same protocol was conducted but without the addition of CalB buffer solution.

### 3.3. Protein Concentration and Enzymatic Activity

For enzyme characterization, protein concentration and enzymatic activity were evaluated for SiO_2_ reference support, free CalB, and biosilicified samples.

Protein concentration was determined according to the Bradford colorimetric method [29], using protein bovine serum albumin as standard. All solutions were prepared by employing ultrapure water.

Enzymatic activity was determined by hydrolysis of pNPB in acetonitrile, at pH 7 and 25 °C according to the protocol described by García-Galán et al. [30]. p-nitrophenol, released as the hydrolysis product of pNPB to the reaction medium was spectrophotometrically measured at 400 nm. In the present work, an activity unit (U) was defined as the amount of enzyme capable to hydrolyse 1 µmol of substrate per minute at pH 7 and 25 °C (Equation (1)).

(1) $$\mathrm{Enzymatic}\;\mathrm{activity}\;\left(U\right)=\frac{µ\mathrm{mol}\;\mathrm{of}\;\mathrm{substrate}\;\mathrm{hydrolysed}}{\mathrm{minute}}$$

The specific activity, as an important measure of enzyme purity, was expressed as µmol of substrate hydrolysed per g of enzyme per min of incubation, being corrected for blank values (Equation (2)).

(2) $$\mathrm{Specific}\;\mathrm{activity}\;\left({U/g}_{\mathrm{biocatalyst}}\right)=\frac{U}{g\;\mathrm{of}\;\mathrm{enzyme}}$$

### 3.4. Immobilization Efficiency

Immobilization efficiency was evaluated with two parameters: protein loading ratio (Equation (3)) and activity yield (Equation (4)).

(3) $$\mathrm{Protein}\;\mathrm{loading}\;\mathrm{ratio}\;\left(\%\right)=\frac{\mathrm{protein}\;\mathrm{loaded}}{\mathrm{protein}\;\mathrm{introduced}}\;\times \;100$$

(4) $$\mathrm{Activity}\;\mathrm{yield}\;\left(\%\right)=\frac{\mathrm{specific}\;\mathrm{activity}\;(\mathrm{immobilized}\;\mathrm{lipase})}{\mathrm{specific}\;\mathrm{activity}\;(\mathrm{free}\;\mathrm{lipase})}\;\times \;100$$

Protein loading ratio (%) was used to compare the amount of enzyme that finally remains in the biocatalyst with the free enzyme. Activity yield (%) equally determined the difference between specific activity in case of the biosilicified enzyme and the free form.

### 3.5. Biocatalytic Esterification Activity

The conversion of valeric acid to ethyl valerate was investigated in order to evaluate the activity of the synthetized biocatalysts. Valeric acid and different alcohols (EtOH, MeOH, iPrOH, BuOH) were used as reaction substrates.

For optimization purposes, different experimental sets were conducted in order to evaluate the biocatalyst load (molar ratio 1:2 of valeric acid to EtOH, 1000 rpm, 40 °C, 2 h in CH batch reactor), the presence of water (1:2 molar ratio valeric acid to EtOH, 12% *m*/*v* of biosilicified enzyme, 1000 rpm, 40 °C, 2 h CH in batch reactor), the stirring rate (different molar ratios of valeric acid to EtOH, 12% *m*/*v* of biosilicified enzymes, 250–1000 rpm, 40 °C, 2 h CH in batch reactor) and the reaction temperature (different molar ratios of valeric acid to EtOH, 12% *m*/*v* of biosilicified enzyme, 1000 rpm, 2 h CH in batch reactor).

In a typical esterification reaction, valeric acid was added to each alcoholic solution at 1:1, 1:2 and 1:3 molar ratios. In all the experiments 12% *m*/*v* of biosilicified lipase was used. Mixtures were placed in a microwave reactor (CEM-Discover, Matthews, NC, USA) or in a Carousel Reaction Station™ (Radley Discovery Technologies, Saffron Walden, UK) for MW or CH experiments, respectively. Esterification was conducted for 2 h in all cases, with continuous temperature measurement and periodic sampling. Blank reactions containing the free CalB were run under identical conditions using comparable enzyme quantities (12% *m*/*v* of not-immobilized CalB).

### 3.6. Evaluation of Biocatalytic Esterification Behaviour

From each conducted esterification reaction, the resulting products were quantified by gas chromatography (GC) analysis in an Agilent 7890 GC model fitted with a capillary Petrocol column (100 m × 0.25 nm × 0.5 μm) and a flame ionization detector (FID) and a N_2_ carrier flow of 30 mL min^−1^. Collected samples were 1:6 diluted in the corresponding alcoholic reaction media and filtrated with a 45 µm syringe filter before injection. The GC program was as follows: oven from room temperature (RT) to 220 °C (40 min), inlet at 250 °C (split mode), and detector at 300 °C.

## 4. Conclusions

Optimised conditions were studied in the esterification of valeric acid by biosilicified enzymes via conventional and microwave-assisted heating, resulting in about 80% of valeric acid conversion to ethyl valerate after 15 min using both methodologies under mild reaction conditions. Comparably, the reuse of the enzyme remained stable after different cycles of esterification via conventional heating, while protein folding (leading to enzyme denaturalisation) seemed to take place under microwave-assisted conditions due to the rapid increase of temperature. It can be established that, in spite of exhibiting similar performances, conventional heating assisted esterification required less energy, resulting in being a simpler procedure for implementation in many industrial fields but also allowing easier reuse of this novel biocatalysts.

## Figures and Tables

**Table 1:** Enzymatic activity, protein loading and activity yield of the analysed systems.

System	Enzymatic Activity (U/g_enzyme_)	Protein Loading Ratio (%)	Activity Yield (%)
Support SiO_2_	-	-	-
Free enzyme	656 ± 14	100	100
Biosilicified lipase (R_0_)	592 ± 13	86	90

**Table 2:** Effect of water presence on the conversion of valeric acid to ethyl valerate and on the specific activity of prepared biocatalysts.

Distilled Water (DW) % *m*/*v*	Conversion to Ethyl Valerate (mol %)	Specific Activity (U/g_biocatalyst_)
5	87	269 ± 10
10	85	301 ± 9
20	84	364 ± 11

Reaction conditions: 1:2 molar ratio valeric acid to EtOH, 12% *m*/*v* of biosilicified enzyme, 1000 rpm, 40 °C, 2 h CH in batch reactor.

**Table 3:** Influence of stirring rate on the conversion of valeric acid to ethyl valerate and on the specific activity of prepared biocatalysts.

Acid-to-Alcohol Ratio	Conversion to Ethyl Valerate (mol %)				Specific Activity (U/g_biocatalyst_)			
	250 rpm	500 rpm	750 rpm	1000 rpm	250 rpm	500 rpm	750 rpm	1000 rpm
1:3	73	76	75	81	478 ± 10	490 ± 11	497 ± 9	501 ± 13
1:2	89	81	87	95				
1:1	92	87	87	90				

Reaction conditions: 12% *m*/*v* of biosilicified enzymes, 250–1000 rpm, 40 °C, 2 h CH in batch reactor.

**Table 4:** Effect of reaction temperature on the conversion of valeric acid to ethyl valerate and specific activity of biocatalysts after esterification.

Acid-to-Alcohol Ratio	Conversion to Ethyl Valerate (mol %)							* Specific Activity (U/g_biocatalyst_)			
	50 °C	45 °C	40 °C	35 °C	30 °C	25 °C	20 °C	50 °C	40 °C	30 °C	20 °C
1:3	84	86	94	88	80	82	83	234 ± 11	485 ± 17	623 ± 14	630 ± 13
1:2	85	87	96	91	84	84	87				
1:1	74	78	76	71	75	71	75				

Conditions for esterification reaction: 12% *m*/*v* of biosilicified enzyme, 1000 rpm, 2 h CH in batch reactor. * To determine the influence of temperature on the specific activity of the catalyst, 12% *m*/*v* of biosilified enzyme was incubated in DW, 1000 rpm, 2 h CH in batch reactor at the different temperatures.

**Table 5:** CH esterification of valeric acid using different alcohols (MeOH, EtOH, iPrOH, BuOH) and different acid-to-alcohol molar ratios.

Entry	System	Alcohol	Acid-to-Alcohol Ratio	Conversion to Alkyl Valerate (mol %)	* Specific Activity (U/g_biocatalyst_)
1 2 3 4	Blank	MeOH EtOH iPrOH BuOH	All	No conversion	-
5 6 7 8	Support SiO_2_	MeOH EtOH iPrOH BuOH	All	No conversion	-
9 10 11	Free enzyme	EtOH	1:1 1:2 1:3	82 82 87	600 ± 12
12	Biosilicified enzyme	MeOH	All	No conversion	-
13 14 15	Biosilicified enzyme	EtOH	1:1 1:2 1:3	81 95 90	640 ± 17
16 17 18	Biosilicified enzyme	iPrOH	1:1 1:2 1:3	57 92 75	194 ± 15
19 20 21	Biosilicified enzyme	BuOH	1:1 1:2 1:3	60 89 77	291 ± 10

Conditions for esterification reaction: 12% *m*/*v* biosilicified enzyme (equal quantity for free enzyme using buffer solution) 1000 rpm, 40 °C, 2 h CH in batch reactor. * To determine the influence of different alcohols on the specific activity of the catalyst, 12% *m*/*v* of biosilificated enzyme was incubated in MeOH, EtOH, iPrOH, BuOH, respectively, 1000 rpm, 40 °C, 2 h CH in batch reactor.

**Table 6:** Reusability studies of biosilicified enzymes for esterification reactions with different alcohols under CH.

Reuse	Conversion to Ethyl Valerate (mol%)					Specific Activity (U/g_biocatalyst_)				
	MeOH	EtOH	IPrOH	BuOH	Free Enzyme	MeOH	EtOH	iPrOH	BuOH	Free Enzyme
R_0_	-	95 ± 8	92 ± 7	89 ± 8	82 ± 6	418 ± 14	506 ± 16	508 ± 14	427 ± 12	352 ± 12
R_1_	-	88 ± 7	42 ± 7	80 ± 9	75 ± 8	207 ± 12	505 ± 12	400 ± 19	307 ± 14	307 ± 16
R_2_	-	94 ± 6	-	70 ± 7	68 ± 5	-	415 ± 18	367 ± 15	270 ± 12	301 ± 15
R_3_	-	92 ± 8	-	69 ± 9	60 ± 8	-	400 ± 14	-	254 ± 17	272 ± 9
R_4_	-	93 ± 6	-	60 ± 7	61 ± 6	-	403 ± 11	-	207 ± 16	260 ± 13

Reaction conditions: 12% *m*/*v* biosilicified enzyme (equal quantity for free enzyme using buffer solution), 1000 rpm, 40 °C, 2 h CH in batch reactor.

**Table 7:** MW-assisted esterification of valeric acid using different alcohols (MeOH, EtOH, iPrOH, BuOH) and different acid-to-alcohol molar ratios.

Entry	System	Alcohol	Acid-to-Alcohol Ratio	Conversion to Alkyl Valerate (mol %)	Specific Activity (U/g_biocatalyst_)
1 2 3 4	Blank	MeOH EtOH iPrOH BuOH	All	No conversion	-
5 6 7 8	Support SiO_2_	MeOH EtOH iPrOH BuOH	All	No conversion	-
9 10 11	Free enzyme	EtOH	1:1 1:2 1:3	70 75 71	270 ± 13
12	Biosilicified enzyme	MeOH	All	No conversion	-
13 14 15	Biosilicified enzyme	EtOH	1:1 1:2 1:3	58 82 73	376 ± 7
16 17 18	Biosilicified enzyme	IPrOH	1:1 1:2 1:3	39 40 37	300 ± 13
19 20 21	Biosilicified enzyme	BuOH	1:1 1:2 1:3	79 81 80	337 ± 5

Reaction conditions: 12% *m*/*v* biosilicified enzyme (equal quantity for free enzyme using buffer solution), 100 W, 40 °C, 1 h MW radiation.

**Table 8:** Effect of reaction time on MW and CH conducted esterification of valeric acid under the main optimised reaction conditions.

Methodology	Conversion to Ethyl Valerate (mol %)			Specific Activity (U/g_biocatalyst_)		
	15 min	30 min	60 min	15 min	30 min	60 min
MW	80	80	82	394 ± 18	384 ± 16	357 ± 10
CH	86	90	90	545 ± 14	495 ± 11	460 ± 11

Reaction conditions: molar ratio 1:2 of valeric acid to EtOH, 12% *m*/*v* biosilicified enzyme, 100 W (MW), 40 °C (CH).

**Table 9:** Reusability studies of biosilicified enzymes in the esterification reactions using MW radiation.

Reuses	Conversion to Ethyl Valerate (mol %)	Specific Activity (U/g_biocatalyst_)
R_0_	82 ± 9	357 ± 14
R_1_	66 ± 4	310 ± 9
R_2_	51 ± 6	285 ± 11
R_3_	35 ± 8	210 ± 12

Reaction conditions: molar ratio 1:2 of valeric acid to EtOH, 12% *m*/*v* biosilicified enzyme, 100 W, 40 °C, 1 h MW radiation.
